# Feasibility and Safety of the Routine Distal Transradial Approach in the Anatomical Snuffbox for Coronary Procedures: The ANTARES Randomized Trial

**DOI:** 10.3390/jcm12247608

**Published:** 2023-12-11

**Authors:** Łukasz Koziński, Zbigniew Orzałkiewicz, Alicja Dąbrowska-Kugacka

**Affiliations:** 1 Department of Cardiology, Chojnice Specialist Hospital, Lesna 10, 89-600 Chojnice, Poland; 2 Department of Cardiology and Electrotherapy, Medical University of Gdansk, Smoluchowskiego 17, 80-214 Gdansk, Poland

**Keywords:** transradial access, snuffbox, distal transradial approach, radial artery occlusion, coronary angiography, vascular ultrasonography

## Abstract

The distal transradial approach (dTRA) through the anatomical snuffbox is hypothesized to offer greater benefits than the conventional transradial access (cTRA) for patients undergoing coronary procedures. Our goal was to assess the safety and efficacy of dTRA. Out of 465 consecutive Caucasian patients, 400 were randomized (1:1) to dTRA or cTRA in a prospective single-center trial. Clinical and ultrasound follow-ups were obtained at 24 h and 60 days post-procedure. The primary combined endpoint consisted of access crossover, access-related complications, and major adverse cardiovascular events (MACE). Secondary endpoints included clinical success endpoints (puncture success, crossover, and access time), access-site complications endpoints, and MACE at 60 days. The primary endpoint was significantly higher in the dTRA [odds ratio (OR): 2.31, 95% confidence interval (CI): 1.38–3.86, *p* = 0.001]. Clinical success endpoints, namely crossover (10% vs. 3.5%, *p* < 0.05) and access-time [median: 140s (85–322) vs. 80s (58–127), *p* < 0.001], did not favor the dTRA, despite a similar success rate in radial artery puncture between the dTRA and cTRA (99.5% vs. 99%). Radial artery spasm (19% vs. 4.5%, *p* < 0.0001), physical discomfort during access, and transient thumb numbness after the procedure occurred more frequently with the dTRA. However, early (2.5% vs. 4.5%, *p* = 0.41) and mid-term (2.5% vs. 3%, *p* = 0.98) forearm radial artery occlusion rates were comparable between the dTRA and cTRA. Randomization to the dTRA, lower forearm radial pulse volume, higher body mass index, and lower body surface area independently predicted the primary endpoint in multivariate analysis. In the interaction effect analysis, only diabetes increased the incidence of the primary endpoint with the dTRA (OR: 18.67, 95% CI: 3.96–88.07). The dTRA was a less favorable strategy than cTRA during routine coronary procedures due to a higher incidence of arterial spasm and the necessity for access crossover. The majority of local complications following the dTRA were clinically minor complications. Individuals with diabetes were particularly susceptible to complications associated with the dTRA.

## 1. Introduction

The transradial approach (TRA) has become the standard strategy for both urgent and elective coronary procedures in most countries [1,2,3]. This approach offers numerous advantages over transfemoral access, including a reduction of mortality, complications, and cost, while also enhancing patient comfort [4,5]. Some issues remain unresolved, e.g., postprocedural radial artery occlusion (RAO) is still the Achilles’ heel.

Innovatively, Kiemeneij [6] has revitalized radial artery (RA) access by utilizing the anatomical snuffbox. This novel approach is believed to combine the practicality of the conventional transradial approach (cTRA) with even greater safety, owing to the radial artery’s connection with the arterial superficial palmar arch, its more superficial course, and enhanced compressibility. Numerous studies concerning the distal transradial approach (dTRA) have been published [6,7,8,9,10,11,12,13,14,15,16,17]. They suggest that this method is feasible and safe in selected patients, but there is limited data regarding the dTRA as an everyday routine. Due to the reduced RA diameter in the snuffbox and its distal tortuosity, several questions emerge regarding the dTRA as a standard practice.

The aim of the present study (ANTARES, distAl vs. coNventional Transradial Access for coRonary procEdures Study) was to assess the feasibility and safety of the dTRA in the anatomical snuffbox as the preferred first-line approach, in comparison with the cTRA, in patients undergoing coronary angiography (CAG) and percutaneous coronary intervention (PCI).

## 2. Material and Methods

### 2.1. Study Design and Population

The ANTARES was a single-center, randomized, controlled, prospective trial conducted in a regional hospital. For a further 4 months (1 November 2022 to 28 February 2023), consecutive, adult, Caucasian patients referred for CAG and/or PCI were screened for eligibility. The exclusion criteria were as follows: ST-elevation myocardial infarction, sudden cardiac arrest, hemodynamic instability, chronic kidney disease (stages 4–5), forearm artery occlusion, previous unsuccessful ipsilateral TRA, unfavorable RA diameter, and ultrasound unavailability. The procedure could be the first or subsequent ipsilateral radial one. Two operators (LK and ZO), with ≥50 cases of snuffbox access experience before the trial started, performed the procedures.

Randomization (1:1) to the distal or conventional TRA group was performed using sealed envelopes and carried out in groups of 50 participants to maintain the same number of patients in both groups throughout the study. All participants provided written informed consent prior to enrollment in the study. Our study protocol was compliant with the standards as described in the Declaration of Helsinki and was approved by the Bioethical Commission at the Regional Medical Chamber (KB–25/18) NCT05982366.

### 2.2. Ultrasonography

Vascular ultrasonography was mandatory because an arterial pulse is often falsely present as a result of blood supply from collaterals, and the manual palpation method can erroneously indicate artery obstruction. It was performed using a Vivid-7 ultrasound (General Electric Healthcare, Chicago, IL, USA) equipped with a linear 10 MHz transducer within 24 h before the procedure and at every follow-up visit. The eligibility criteria consisted of both radial and ulnar artery patency with an RA dimension in the radial fossa of ≥1.8 mm, which was based on our previous experience.

### 2.3. Radial Access

The choice of right or left side procedure was at the operator’s discretion based on manual pulse palpation and ultrasound results. For the right dTRA, the arm was placed in a comfortable, neutral position, parallel to the torso, with the thumb flexed under the other fingers and with the hand slightly flexed. Whereas for the left dTRA, the forearm was shifted to the right half of the body with a roller placed under the elbow for the patient’s convenience and the hand positioned on the right lower abdomen, slightly adducted, with the thumb inside the clenched fist. These preparations provided a more superficial localization of the distal RA within the anatomical snuffbox. During the procedure, the operator stood on the patient’s right side. Conventional TRA was obtained by the operator standing on the chosen access side, the patient’s forearm was positioned with external rotation, and a small gauze roller was placed under the wrist for better exposure of the RA.

The vessel puncture was performed after local anesthesia with 1.0 mL of 2% lidocaine hydrochloride. Vascular ultrasound was not allowed for artery puncture guidance. The preferred RA puncture site for the cTRA was 2 cm proximally to the styloid process of the radial bone, whereas for the dTRA it was proximally in the anatomical snuffbox.

Four types of hydrophilic 10 cm long transradial kits were used: 5-Fr/6-Fr Radifocus^®^ Introducer II and 6-Fr/7-Fr Glidesheath Slender^®^ Introducer (Terumo Corp., Tokyo, Japan). The selection of vascular sheath size was at the operator’s discretion.

RA puncture was performed with a 22-gauge metal needle under a 30–45° angle, preferably using the modified Seldinger anterior wall technique. After obtaining arterial drops of blood, a 0.018″, J-shaped, 45 cm long mini-guidewire was advanced. Then the needle was removed, and a sheath was inserted by threading the guidewire. Five milligrams of verapamil hydrochloride was slowly injected intra-arterially, followed by a 10 mL bolus of 0.9% NaCl, to cause vasodilation. In cases of RA spasm, 0.2 mg of nitroglycerin was given intra-arterially. The mini-guidewire was replaced with a 0.035″, J-shaped, 200 cm wire afterward. Unfractionated heparin (70–100 IU/kg body weight) was administered after coronary artery intubation during angioplasty, while for a diagnostic study, 2500 IU was given at the end of the procedure. After completion of the procedure, the operator removed the sheath and placed a compressive dressing (gauze and bandage) on the puncture site for 4 h after CAG and for 5 h after PCI.

### 2.4. Follow-Up and Study Endpoints

The clinical evaluation and vascular ultrasonography were scheduled at 24 h and 60 days post-procedure. The primary composite endpoint of our trial consisted of access crossover, major adverse cardiovascular events (MACE), and access-related vascular complications. MACE measured 60 days post-procedure was defined as myocardial infarction, stroke, urgent revascularization, and all-cause death. Access-related complications evaluated 24 h after the procedure included RAO at the puncture site, significant hematoma (scored using the EASY classification grade III–V) [18], arteriovenous fistula, pseudoaneurysm, and RA perforation and spasm.

The secondary endpoints included clinical success and access-site complications endpoints, and MACE at 60 days. Clinical success endpoints comprised successful radial artery puncture with the needle, access crossover rate, and duration of access performance (measured from the time of skin puncture with a local anesthetic to the successful sheath insertion confirmed by an outflow of arterial blood). Access-site complications endpoints consisted of the following: all possible vascular complications, the patient’s discomfort evaluated while gaining access, and post-procedural local neuropathy. Radial artery spasm was defined intraprocedurally as difficult manipulation and resistance, or trapping during insertion of the guidewire, sheath, or catheter into the RA, often with accompanying pain. The patients’ physical discomfort at the time of vascular access performance was assessed using a numerical scale (0/1/2/3, associated with no/mild/moderate/severe discomfort, respectively). An ultrasound examination was performed to rule out RAO, arteriovenous fistula, pseudoaneurysm, and RA stenosis.

### 2.5. Statistical Analysis

Clinical, angiographic, and procedural data were prospectively entered into a computerized database. The Shapiro-Wilk test was used to estimate the distribution. Continuous variables were expressed as the mean ± standard deviation (SD) and compared using an unpaired *t*-test or Mann-Whitney *U* test, where appropriate. The non-normally distributed data were expressed as the median together with the interquartile range (IQR). Categorical variables were expressed as absolute or relative frequencies and compared using chi-square analyses or the Fisher exact test, as appropriate to the cell frequencies.

The logistic regression method was applied to identify variables that independently predicted the primary composite endpoint. Predictors with a univariate *p*-value ≤ 0.2 were included in the multivariate logistic regression model, after excluding co-linearity between them. The risk of primary composite endpoint occurrence was expressed as an odds ratio (OR) with a 95% confidence interval (CI). Analysis was performed according to the intention-to-treat and treatment-per-protocol.

To assess which demographic or clinical factors were correlated with a significantly higher risk of the primary endpoint when randomized to the snuffbox group, the interaction effects were analyzed. The statistical significance of the interaction term between the intervention and clinical factors was assessed using multivariate logistic regression. A *p*-value < 0.05 was considered statistically significant. STATA software (version 9.2, StatCorp, College Station, TX, USA) was used to calculate statistics.

### 2.6. Sample Size Calculation

Considering the results of the first 100 patients, a primary event rate of 20% was estimated for patients in both treatment groups: ~2% MACEs, ~7% cross-over rate, ~7% access-related vascular complications at 24 h, and ~4% at 2 months. To obtain 80% statistical power with a 2-sided α = 0.05, about 200 patients in each treatment group (400 in total) were needed to establish a clinically important difference of 10% between the randomized groups.

## 3. Results

### 3.1. Study Population

**Figure 1:**
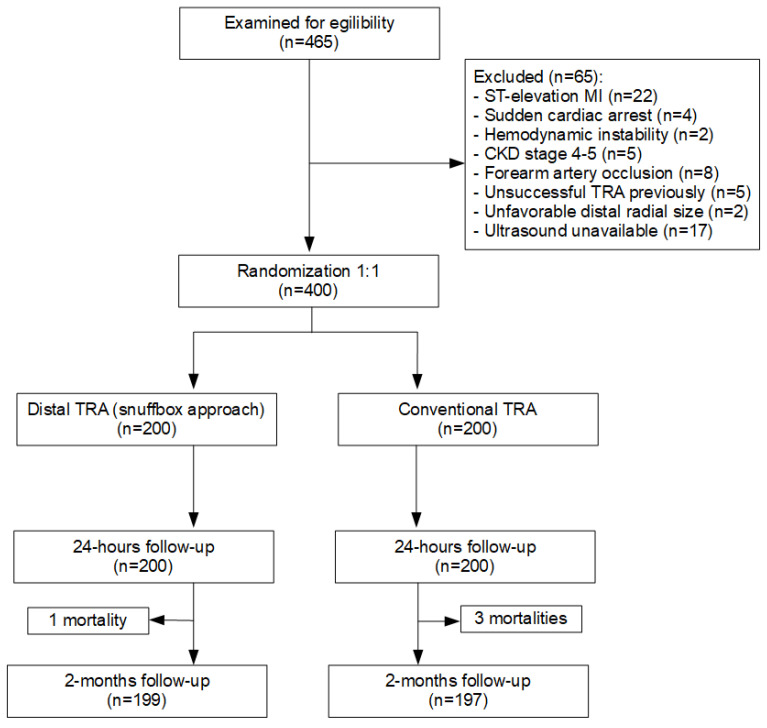
Study flow chart. TRA = transradial approach; CKD = chronic kidney disease; MI = myocardial infarction.

Out of 465 screened patients, a total of 400 were randomized: 200 to the snuffbox group and 200 to the conventional TRA group (Figure 1). Baseline characteristics of patients were matched (Table 1). Approximately a quarter of the patients in each group presented with non-ST elevation acute coronary syndrome.

### 3.2. Procedural Data

The procedural and angiographic data are presented in Table 2. The overall number of CAGs and PCIs, and the frequency of the left-sided access and prior ipsilateral radial route did not differ between the dTRA and cTRA groups. Successful artery puncture was comparable between groups; nevertheless, the success rate of sheath insertion into the RA was significantly lower in the dTRA cohort (90% vs. 98.5%, *p* < 0.001). There were no significant differences in fluoroscopy time, fluoroscopy effective dose, contrast load, or heparin dose.

### 3.3. Primary Endpoint

**Figure 2:**
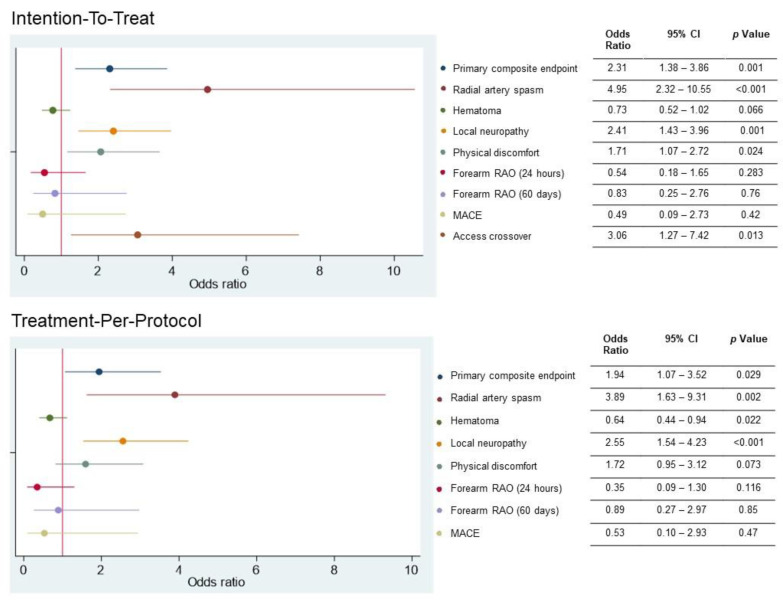
Selected study outcomes for the intention-to-treat and treatment-per-protocol analyses. The primary composite endpoint consisted of access crossover, major adverse cardiovascular events, and access-related vascular complications. RAO = radial artery occlusion; MACE = major adverse cardiac and cerebrovascular events.

In the intention-to-treat analysis, the occurrence of the primary composite endpoint was significantly higher in the dTRA group (53 patients [26.5%]) than in the cTRA (27 patients [13.5%]) (OR: 2.31, 95% CI: 1.38 to 3.85, *p* < 0.001). This was mainly driven by the incidence of crossover and access-related complications. Similarly, in the treatment-per-protocol analysis, the occurrence of the primary endpoint was higher when the dTRA was performed (OR 1.94, 95% CI: 1.07 to 3.52, *p* = 0.029) (Figure 2).

### 3.4. Clinical Success Endpoints

The success rate of radial artery puncture with the needle was comparable between the dTRA and cTRA (99.5% vs. 99%). Access crossover occurred in 10% and 3.5% in the dTRA and cTRA groups, respectively (*p* < 0.05). While the causes for access change in an ineffective cTRA were clear-cut (Table 2), an access crossover in the snuffbox group was related to spasm and distal tortuosity of RA. In nine of 20 patients from the dTRA group, the 0.018″ wire could not pass through the RA. The median time of successful primary vascular access performance was 140 s (IQR 85-322 s) in the dTRA cohort, and it took about 60 s longer than in the cTRA cohort (80 s [IQR 58-127 s], *p* < 0.001).

### 3.5. Access-Site Complications Endpoints

Clinical and ultrasound outcomes at both observation periods are provided in Table 3 and Table 4. RA spasms occurred in 19% of patients in the dTRA group and 4.5% of the cTRA group (*p* < 0.0001). Arterio-venous fistulae were detected by ultrasonography in four dTRA patients: two were treated with compression, one fistula closed spontaneously, and one persisted (but with inappreciable flow). The incidence of hematomas, pseudoaneurysms, and RA perforation did not differ between groups. No cases of RA stenosis, active external bleeding from the puncture site, or hand ischemia were observed.

Physical discomfort during vascular access occurred in 12% of the dTRA group and only in 3% of the cTRA group (*p* < 0.01). Local neuropathy, in the form of thumb numbness persisting up to a few hours, occurred in 29% of patients after the dTRA, and in 14.5% after cTRA (*p* < 0.001). After 2 months no persistent neuropathy was observed in the dTRA cohort.

The incidence of RAO was comparable between the cohorts in both follow-up periods. Vascular ultrasound performed 24 h post-procedure in the dTRA showed 11 (5.5%) RAOs at the puncture site with four (2%) concomitant proximal RAOs at the wrist. At the 2-month follow-up, the rate of distal RAO decreased to 5 (2.5%). The overall number of forearm RAOs was five (2.5%), but two of these were found in previously unobstructed arteries despite preserved flow at the snuffbox.

The occurrence of RAO at the puncture site 24 h after the cTRA was 4.5% (nine patients) and concomitant distal occlusion was present in eight of them. All these patients had successful radial procedures. At the 2-month follow-up, the number of proximal RAOs decreased to 6 (3%), but two of them occurred in prior patent arteries. In four of the six cases, distal RA in the snuffbox was also obstructed.

### 3.6. Exploratory Outcomes

Table 5 shows univariate and multivariate predictors of the primary endpoint. In univariate analysis, randomization to the dTRA, lower RA pulse volume, female sex, lower height, weight, and body surface area (BSA), and previous ipsilateral TRA access were significantly related to the primary composite endpoint. In multivariable analysis, randomization to the dTRA, lower forearm RA pulse volume, lower BSA, and higher body mass index (BMI) were independent predictors of the primary combined endpoint.

There was a significantly higher risk of the primary endpoint in patients with diabetes randomized to the snuffbox group (OR: 18.67, 95% CI: 3.96 to 88.07) than in patients without diabetes (OR: 2.04, 95% CI: 1.05 to 3.94); *p* < 0.05 for the interaction analysis.

## 4. Discussion

Our trial was one of the few that confronted the utility of the snuffbox and classic transradial approach for coronary procedures in a randomized fashion. Our results revealed that the dTRA is satisfactorily feasible in terms of the first-line strategy. However, as compared with the cTRA, it was burdened with a greater risk of access failure, more frequent minor access complications (mainly spasm and thumb neuropathy), caused greater patient discomfort during access, and was more time-consuming. Nevertheless, it should be emphasized that the majority of these local complications (such as thumb numbness and hematomas) were clinically insignificant and/or minor complications. Randomization to the dTRA, lower forearm RA pulse volume, higher BMI, and lower BSA were identified as independent predictors of the primary endpoint, consisting of access crossover, access-related vascular complications, and MACE. Additionally, in patients with diabetes, the risk of the primary composite endpoint was nine times higher when randomized to the dTRA than in non-diabetics.

We used both right- and left-sided dTRA. Left-sided dTRA is more convenient for the operator, especially in patients with right subclavian artery tortuosity. The refinement of the right-sided cTRA into dTRA may particularly facilitate coronary procedures in obese patients and those with upper limb dysfunction, where external rotation of the forearm is vexatious. Despite numerous enthusiastic reports on the dTRA, to date, there is no clear clinical evidence supporting the routine use of snuffbox access for coronary procedures in terms of feasibility and safety in a randomized comparison with the cTRA.

### 4.1. Access Failure

Kiemeneij published a report concerning the snuffbox approach for coronary interventions and shared his experience with an 11% crossover in a selected group of 70 out of 118 pre-screened patients [6]. In the first randomized study comparing both approaches for diagnostic angiography of 200 patients, Koutouzis et al. [9] indicated a significant difference in access failure: 30% in the dTRA vs. 2% in the cTRA (*p* < 0.001). In another randomized trial [10], comprising 205 patients, access crossover was 5% in the dTRA and 4% in the cTRA (*p* = 0.47). In a retrospective comparison of both approaches [11], the crossover rate did not differ between groups and equaled 2.4% in the cTRA and 2.0% in the dTRA; however, ultrasound guidance was available in one-third of the dTRA group. Other registries, including from 47 to a maximum of 435 enrolled patients, showed primary access failure and crossover rates from 23% (23/100 consecutive patients referred for CAG) to 0% in a group of 47 selected patients presenting for elective CAG [12,13,14]. One study retrospectively evaluated the feasibility of the dTRA in the context of ST-elevation myocardial infarction, revealing a low crossover rate of 7% (10 of 138 patients with a well-palpable pulse) [15]. Although the femoral artery is traditionally selected for complex PCI with 7-Fr access, the dTRA was also tested in that setting because the smaller radial size might limit the new method. Gasparini et al. reported a 17% (7/41) rate of access failure when using 7-Fr Glidesheath Slender^®^ sheaths [16]. In the vast majority of the mentioned papers, the success rate of artery puncture with a needle was higher than radial cannulation with a sheath.

Our findings support the evidence that the routine snuffbox approach is effective for 90% of patients but falls significantly short of the success rate of 96.5% achieved with the cTRA. The main drawback limiting the vessel access was the vasospasm and tortuosity of the distal part of the RA. Others also listed spasm, tortuosity, and failed artery puncture among the main causes of crossing over [6,9,10,13]. In some cases, we experienced decreased RA pulse filling after subcutaneous anesthesia, which restricted the localization of the puncture site and increased the number of attempts. We are convinced that anesthetic infiltration into a small space such as the snuffbox might result in vessel spasm. Moreover, each attempt at vessel puncture may cause needle-mediated arterial spasm. If advancing with a standard wire into the forearm part of the RA fails, a 0.014″ coronary wire might be useful [14,17]. It appears logical that the frequency of access failure after routine dTRA cannot be lower than after cTRA because it shares the same restrictions (e.g., upper limb arterial system anomaly) and additionally specific snuffbox limitations (e.g., smaller diameter, spasm, tortuosity). Presumably, the routine use of ultrasound guidance may improve the percentage of successful arterial punctures and first passes and reduce the number of puncture attempts.

### 4.2. Access Duration

In the current study, the median time of snuffbox access was approximately 60 s longer than in the conventional arm. Other comparative studies also confirm the longer access duration in the dTRA; in one study about 10 s longer (*p* = 0.008), and in another 129 s longer (*p* < 0.001) [9,10]. Some observational studies reported that the cannulation time was dependent on the operator’s dTRA experience [14,17]. Despite the slightly longer time to obtain the snuffbox access, it does not appear to have clinical relevance in the non-urgent scenario.

### 4.3. Radial Artery Occlusion

Postprocedural RAO remains the most relevant drawback of the TRA. The latest randomized clinical trials, highlighting the prevention improvements, estimate that RAO frequency after the cTRA has decreased from 7.7% to 3.7% at highly experienced centers [19]. In our cTRA cohort, we observed a 4.5% rate of acute forearm RAO, and this complication was reduced to 3% after 60 days.

The advantage of the dTRA is that even in the case of prolonged snuffbox compression, the blood flow is still maintained through the superficial palmar branch of the RA to the superficial palmar arch. This encourages the use of new access because it potentially spares RA patency at the traditional point of puncture in the case of distal RAO. There is scarce data regarding RAO after the dTRA. Initial, and mostly small studies, estimate the rate of in-hospital RAO at 0% to 7% [6,9,11,13,16,17,20]. Another issue is the method used to assess this crucial complication. Only ultrasound guidance allows precise estimation of some RA access complications, such as RAO. In our trial, the number of RAOs did not differ between the two approaches: 5.5% acute distal and 2.5% acute forearm RAO after the snuffbox approach. After 60 days, ultrasound imaging revealed 2.5% distal and 2.5% forearm RAO. We observed spontaneous recanalization in 54% (6/11) of distal RAOs and 40% (2/5) of forearm RAOs. Nevertheless, two new forearm RAOs appeared in prior patent RA. All patients diagnosed with RAO were asymptomatic.

Despite the more popular choice of smaller non-slender sheaths in the dTRA, because the operators were biased, the RAO rates were comparable between both groups in both follow-up periods. Given the smaller diameter of the distal RA, the use of slender sheaths provides a lower sheath-to-artery ratio and may therefore be one of the key factors in reducing the incidence of RAO after the dTRA.

There is no certainty whether RAO is a consequence of artery wall trauma or more of a thrombosis. The RAO prevention methods, e.g., anticoagulation, patent hemostasis, and shortening of hemostatic compression, should have paramount importance even when snuffbox access is performed. Given the low event rate of RAO, a larger trial is needed to verify the concept of maintaining forearm RA patency after the dTRA and the impact of additional preventive techniques.

### 4.4. Other Access-Related Complications

The hypothesis concerning decreased non-RAO access-related complications after the dTRA in comparison with the cTRA was not confirmed in our trial. Data from the literature demonstrated a secure profile and showed a very low incidence of minor access bleeding (from 0% to 10.1%) [11], low occurrence of minor hematoma (0% to 7.4%) [9,11,15,17], and no major bleeding and hematomas. By contrast, in our study population, minor hematomas occurred in 20% of patients, with a similar frequency in the dTRA and cTRA groups. Some attention must be paid to the previously unperceived RA spasm, which occurred significantly more often after the dTRA (19%) than after the cTRA (4.5%), and several times more often than in other papers [9,10]. As already mentioned, distal RA anatomy may be conducive to provoking spasms during RA access, which appears to be the key point for a successful snuffbox procedure. Arterio-venous fistula has been reported sporadically within anatomic snuffbox [13], despite small study cohorts; however, the proximity of the cephalic vein may favor their formation. We experienced a 2% occurrence rate of this rare complication. Ultrasound appears to be the sole tool for detecting arterio-venous fistulae, and prolonged compression in anatomical snuffbox can provide effective therapy at its early stage.

Our study appears to be the first to measure patient comfort at the time of RA access. In this aspect, the snuffbox route turned out to be inferior to the cTRA. Although 88% of patients had no inconvenience, others were exposed to physical discomfort. The RA anatomy might play a crucial role in this issue. Contrary to the literature [6,7,9,10,11,12,13,14,15,16,17], approximately one-third of patients reported transient thumb numbness, which usually persisted a few hours post-procedure. Subcutaneous anesthesia, or less likely mechanical irritation by a sheath, could affect the sensory branch of the radial nerve that supplies the thumb and accompanies the vessels in the snuffbox. Thumb ischemia is an unlikely cause of numbness because the patients had sufficient blood supply from other collaterals and other signs of ischemia were absent. It should be emphasized that the numbness was transient and without any sequelae. Sixty days post-procedure we did not notice local infections associated with the access site, no evidence of inflammation or necrosis of the bone base of the anatomical snuffbox, nor hand ischemia, dysfunction, or neuropathy.

### 4.5. Exploratory Outcomes

In our study, randomization to the dTRA, lower forearm RA pulse volume, lower BSA, and higher BMI were independent predictors of the primary endpoint. Some of these factors may generate difficulties in localization and puncture of the artery or reflect unfavorable small RA size. The treatment-per-protocol analyses also confirmed a significantly higher incidence of the primary combined endpoint, RA vasospasm, and temporary neuropathy in the dTRA group. Interestingly, in the interaction effect analysis, we found that diabetics are prone to have an almost nine-fold greater risk of the primary endpoint when randomized to the dTRA than non-diabetics. This revelation is not surprising, because diabetes is a predictor of RA vasospasm, and this issue may play an even greater role when the snuffbox approach is chosen. The calcification of the walls of the smaller and tortuous distal RA found in diabetics can also reduce the success of the procedure and favor the complications.

### 4.6. Study Limitations

This was a single-center study involving only two experienced radial operators. Our study protocol assumed identical hemostasis time adopted from classic radial access. Other authors’ reports show that the compression time should be much shorter with the dTRA [7,8,9,10,11] and that it potentially contributes to the decreased frequency of RAO. Low-dose heparin (2500 UI) used after the diagnostic procedure could affect the frequency of RAO; currently, according to numerous studies, the standard dose of 5000 UI is widely used. The relatively small sample size and the small number of events (RAO) increase the risk of bias and limit the applicability of this study. The success rate of the dTRA can improve with operator experience because a learning curve exists, as well as with the use of ultrasound for radial access as the standard of care. We used slender sheaths only in a few cases. It is possible that the smaller external dimensions of slender sheaths may increase the distal RA access-site success and reduce some complications (such as RAO) due to a lower sheath-to-artery ratio and thus less provocation of arterial spasm and mechanical wall trauma. The ultrasonographer was not blinded to the patients’ random allocation. We did not use ultrasound guidance during access performance, which may contribute to an increased rate of successful access, shorter access time, and fewer vascular complications [21]. The follow-up duration was short, but the observation periods in the literature were limited to in-hospital stay or one month. Study results are for Caucasians and may differ among Asians due to the different sizes of the radial arteries.

## 5. Conclusions

Distal TRA was a less favorable strategy than the cTRA as a routine approach for coronary angiography and angioplasty due to a higher incidence of arterial spasm and the necessity for access crossover. The majority of local complications following the dTRA (such as thumb numbness and hematomas) were clinically insignificant/minor complications. Diabetics were especially prone to complications of the dTRA.

## Figures and Tables

**Table 1:** Baseline patient clinical characteristics.

	Snuffbox Group n = 200	Conventional Group n = 200	*p*-Value
Demographics			
Age, years; M (IQR)	67.0 (61.0–73.5)	66.7 (60.0–75.0)	0.98
Female; N (%)	80 (40.0)	79 (39.5)	0.92
Height, m; M (IQR)	1.67 (1.60–1.74)	1.68 (1.60–1.75)	0.45
Weight, kg; M (IQR)	82.5 (70–96)	82.7 (72–94)	0.47
Body mass index, kg/m^2^; M (IQR)	29.7 (26–34)	28.4 (26–32)	0.12
Risk factors and comorbidities			
Hypertension; N (%)	161 (81)	154 (77)	0.39
Diabetes mellitus; N (%)	64 (32)	67 (34)	0.75
Dyslipidemia; N (%)	132 (66)	125 (63)	0.46
Smoking; N (%)	93 (47)	89 (45)	0.69
Obesity (BMI > 30); N (%)	94 (47)	81 (41)	0.19
COPD; N (%)	38 (19)	40 (20)	0.80
Peripheral artery disease; N (%)	21 (11)	16 (8)	0.39
Previous MI; N (%)	38 (19)	45 (23)	0.39
Previous stroke; N (%)	18 (9)	8 (4)	0.07
Previous CABG; N (%)	8 (4)	11 (6)	0.64
Previous PCI; N (%)	54 (27)	58 (29)	0.65
Atrial fibrillation; N (%)	16 (8)	23 (12)	0.24
Indication for procedure			
Stable CAD; N (%)	127 (64)	112 (56)	0.13
Unstable angina; N (%)	31 (16)	37 (19)	0.43
NSTEMI; N (%)	21 (11)	25 (13)	0.53
Heart failure; N (%)	7 (4)	15 (8)	0.13
Ventricular arrhythmia; N (%)	6 (3)	5 (3)	1.00
Planned heart valve/aorta surgery; N (%)	8 (4)	6 (3)	0.79
Periprocedural oral medication			
Aspirin; N (%)	198 (99.0)	197 (98.5)	1.0
ADP inhibitor; N (%)	134 (67.0)	125 (62.5)	0.35
DOAC (unstopped therapy); N (%)	0	1 (0.5)	1.0
Laboratory data			
Hemoglobin, g/dL; M (IQR)	13.9 (12.9–14.8)	14.0 (13.1–15.0)	0.44
Platelets, K/µL; M (IQR)	230 (190–284)	226 (184–271)	0.21
eGFR, mL/min; M (IQR)	83 (68–100)	85 (68–107)	0.48
Echocardiography & Ultrasonography			
LVEF, %; M (IQR)	56 (50–60)	56 (45–60)	0.30
Forearm radial artery lumen diameter, mm; M (IQR)	2.50 (2.2–2.7)	2.45 (2.2–2.7)	0.61
Distal radial artery lumen diameter, mm; M (IQR)	2.20 (2.0–2.4)	2.20 (1.9–2.4)	0.66

Data presented are median (IQR) or N (%). Continuous variables were compared using the unpaired *t*-test or Mann-Whitney *U* test. Categorical variables were compared using chi-square analyses or the Fisher exact test. ADP = platelet adenosine diphosphate receptor; BMI = body mass index; CABG = coronary artery bypass grafting; CAD = coronary artery disease; COPD = chronic obstructive pulmonary disease; DOAC = direct oral anticoagulants; eGFR = estimated glomerular filtration rate; IQR = interquartile range; LVEF = left ventricular ejection fraction; M = median; MI = myocardial infarction; N = number; NSTEMI = non ST-segment elevation myocardial infarction; PCI = percutaneous coronary intervention.

**Table 2:** Procedural and angiographic data.

	Snuffbox Group n = 200	Conventional Group n = 200	*p*-Value
Prior ipsilateral TRA; N (%)	41 (20.5)	49 (24.5)	0.34
Left-sided TRA; N (%)	82 (41)	78 (39)	0.68
Successful artery puncture; N (%)	199 (99.5)	198 (99.0)	1.0
No. of RA puncture before wiring; N (%)			
1	161 (80.5)	170 (85.0)	0.18
2	30 (15)	24 (12)	
≥3	8 (4)	4 (2)	
Successful sheath insertion; N (%)	180 (90.0)	197 (98.5)	<0.001
Access performance time, sec; M (IQR)	140 (85–322)	80 (58–127)	<0.001
Discomfort at the time of vascular access; N (%)			
0—no discomfort	176 (88)	194 (97)	0.02
1—mild discomfort	13 (6.5)	3 (1.5)	
2—moderate discomfort	11 (5.5)	3 (1.5)	
3—severe discomfort	0	0	
Access-site crossover; N (%)	20 (10.0)	7 (3.5)	<0.05
Ipsilateral cTRA	15 (7.5)	-	<0.05
Contralateral cTRA	3 (1.5)	5 (2.5)	
Femoral	2 (1)	2 (1)	
Cause of access crossover; N (%)			
Failed artery puncture	1 (0.5)	2 (1.0)	1.0
Radial spasm and/or dissection	19 (9.5)	3 (1.5)	0.001
Anomalous origin of the RA	0	2 (1)	0.48
Arterial sheath size; N (%)			
5-Fr	117 (59)	72 (36)	<0.0001
Including 6-Fr GS	10 (5)	0	
6-F	83 (42)	128 (64)	
Including 7-Fr GS	1 (0.5)	0	
Procedure; N (%)			
CAG only	134 (67)	123 (61.5)	0.25
PCI only	24 (12)	30 (15)	0.38
CAG and PCI	42 (21)	47 (23.5)	0.55
Guiding catheter size; N (%)			
5-Fr	22 (11)	12 (6)	<0.01
6-Fr	43 (21.5)	65 (32.5)	
7-Fr	1 (0.5)	0	
Extent of CAD; N (%)			
No changes	48 (24.0)	47 (23.5)	0.27
Nonobstructive CAD	38 (19.0)	17 (8.5)	
1-VD	32 (16.0)	43 (21.5)	
2-VD	37 (19)	40 (20)	
3-VD or LMD± any vessel	45 (23)	53 (27)	
Coronary artery treated; N (%)			
Left anterior descending artery	33 (16.5)	31 (15.5)	0.30
Left circumflex artery	13 (6.5)	18 (9.0)	
Right coronary artery	20 (10)	26 (13)	
Left main coronary artery	0	2 (1)	
Angioplasty type; N (%)			
Drug-eluting stent	58 (29.0)	65 (32.5)	0.46
DEB/POBA	5 (2.5)	10 (5.0)	
Unsuccessful PCI	3 (1.5)	2 (1.0)	0.86
Stents implanted per PCI; M (IQR)	1.03 (0–2)	0.88 (0–2)	0.52
Unfractionated heparin; N (%)			
2500 IU	131 (66)	120 (60)	0.26
≥5000 IU	67 (34)	78 (39)	0.25
Fluoroscopy time, min; N (%); M (IQR)	3.6 (2.0–7.9)	4.0 (2.0–7.3)	0.94
Fluoroscopy effective dose, mGy; M (IQR)	203 (111–438)	196 (99–416)	0.64
Contrast volume, mL; M (IQR)	40 (25–87)	47 (30–97)	0.18
Total procedure time, min; M (IQR)	18.9 (11.0–34.1)	16.5 (9.7–31.5)	0.09

Data presented are median (IQR) or N (% of the total snuffbox or conventional group). Continuous variables were compared using the unpaired *t*-test or Mann-Whitney *U* test. Categorical variables were compared using chi-square analyses or the Fisher exact test. CAD = coronary artery disease; CAG = coronary angiography; cTRA = conventional transradial approach; DEB = drug eluting balloon; Fr = French; GS = Glidesheath Slender^®^ introducer (Terumo); IQR = interquartile range; IU = international unit; LMD = left main coronary artery disease; M = median; N = number; PCI = percutaneous coronary intervention; POBA = plain old balloon angioplasty; RA = radial artery; Sec = second; TRA = transradial approach; VD = coronary vessel disease.

**Table 3:** Clinical and ultrasonic outcomes within 24-h follow-up.

	Snuffbox Group n = 200	Conventional Group n = 200	*p*-Value
Radial artery spasm	38 (19.0)	9 (4.5)	<0.0001
Hematoma *			
No hematoma	160 (80)	151 (76)	0.11
Grade I	33 (16.5)	31 (15.5)	
Grade II	7 (3.5)	16 (8.0)	
Grade III	0	2 (1)	
Grade IV	0	0	
Grade V	0	0	
Active external bleeding from puncture site	0	0	1.0
Hand ischemia	0	0	1.0
Radial artery perforation	1 (0.5)	1 (0.5)	1.0
Radial artery stenosis **	0	0	1.0
Pseudoaneurysm	0	1 (0.5)	1.0
Arteriovenous fistula	4 (2)	0	0.13
RAO at puncture site	11 (5.5)	9 (4.5)	0.82
RAO at second site	5 (2.5)	9 (4.5)	0.41
Forearm RAO	5 (2.5)	9 (4.5)	0.41
Prolonged radial compression	0	1 (0.5)	1.0
Local neuropathy (thumb numbness)	58 (29)	29 (14.5)	<0.001
BARC bleeding type 2, 3, 5	0	1 (0.5%)	1.0

Data presented are N (%). Categorical variables were compared using chi-square analyses or the Fisher exact test. BARC = Bleeding Academic Research Consortium scale; RAO = radial artery occlusion; * according to EASY (Early Discharge After Transradial Stenting of Coronary Arteries Study) hematoma scale; ** defined as an increase in peak systolic velocity > 2× the baseline value on Doppler-ultrasound.

**Table 4:** Clinical and ultrasonic outcomes after 2 months follow-up.

	Snuffbox Group n = 199	Conventional Group n = 197	*p*-Value
Radial artery stenosis	1 (0.5)	1 (0.5)	0.48
RAO at puncture site	5 (2.5)	6 (3.0)	0.98
RAO at second site	5 (2.5)	5 (2.5)	0.76
Forearm RAO	5 (2.5)	6 (3.0)	0.98
Local neuropathy	0	1 (0.5)	0.99
Puncture-site infection	0	0	1.0
Bone base inflammation/necrosis *	0	0	1.0
Hand ischemia	0	0	1.0
BARC bleeding type 2, 3, 5	1 (0.5)	1 (0.5)	1.0
MACE	2 (1)	4 (2)	0.68
Myocardial infarction	0	0	1.0
Stroke	0	0	1.0
Urgent revascularization	1 (0.5)	1 (0.5)	1.0
Death (any cause)	1 out of 200 (0.5)	3 out of 200 (1.5)	0.62

Data presented are N (%). Categorical variables were compared using chi-square analyses or the Fisher exact test. * the bone base under the puncture site of a radial artery; BARC = Bleeding Academic Research Consortium scale; RAO = radial artery occlusion; MACE = major adverse cardiac and cerebrovascular events.

**Table 5:** Univariate and multivariate predictors of the primary composite endpoint in the study population.

	Odds Ratio	95% CI	*p*-Value
Univariate Analysis			
Randomization to dTRA	2.31	1.38–3.86	0.001
Forearm RA pulse volume	0.37	0.23–0.59	<0.001
Snuffbox RA pulse volume	0.44	0.26–0.74	0.002
Gender	0.49	0.30–0.80	0.005
Height	0.006	0.0004–0.094	<0.001
Weight	0.97	0.96–0.99	0.003
BSA	0.11	0.03–0.36	<0.001
Dyslipidemia	0.61	0.37–1.01	0.055
No. of RA punctures	0.55	0.28–1.06	0.076
2nd ipsilateral TRA	0.33	0.13–0.87	0.025
Hypertension	0.65	0.37–1.13	0.128
Previous myocardial infarction	0.62	0.32–1.21	0.159
Diabetes	0.68	0.39–1.17	0.163
Multivariate analysis			
Randomization to dTRA	2.89	1.65–5.08	<0.001
Forearm RA pulse volume (higher)	0.42	0.23–0.76	0.004
BMI	1.08	1.00–1.17	0.04
BSA	0.044	0.004–0.467	0.01

The primary composite endpoint consisted of access crossover, major adverse cardiovascular events (MACE), and access-related vascular complications. BMI = body mass index; BSA = body surface area; dTRA = distal transradial approach; CI = confidence interval; RA = radial artery; TRA = transradial approach.

## Data Availability

The data underlying this article will be shared on reasonable request to the corresponding author.
